# Molecular Determinants of Bone Plasticity Regeneration After Trauma: Forensic Consequences

**DOI:** 10.3390/ijms26157184

**Published:** 2025-07-25

**Authors:** Sorin Hostiuc, Ionut Negoi, Mihnea Costescu, Costel Siserman

**Affiliations:** 1 Department of Legal Medicine and Bioethics, Carol Davila University of Medicine and Pharmacy, 020021 Bucharest, Romania; 2 Department of Surgery, Carol Davila University of Medicine and Pharmacy, 020021 Bucharest, Romania; negoiionut@gmail.com; 3 Department of Pharmacology, Carol Davila University of Medicine and Pharmacy, 020021 Bucharest, Romania; mihnea.costescu@umfcd.ro; 4 Department of Legal Medicine, University of Medicine and Pharmacy, 400006 Cluj-Napoca, Romania; cvsiserman@gmail.com

**Keywords:** molecular determinants, bone, molecular signatures, forensic science

## Abstract

Bone tissue is one of the most remarkable examples of biological plasticity within the human body, with a high regenerative capacity and adaptation following traumatic injuries. This process is conducted through a series of complex and interlinked molecular mechanisms, which will be summarized in this study. The temporal progression of bone healing follows relatively predictable phases, characterized by variation in the concentration and/or activity of biomolecules such as BMP, VEGF, MMPs. The molecular understanding of bone plasticity and regeneration has potentially significant implications in forensic sciences. They were not extensively studied and implemented in practical, forensic environments, mainly due to their high costs and limited availability. However, they have potential uses in areas, such as the interpretation of skeletal trauma, the estimation of the post-traumatic intervals, the postmortem interval, or the differentiation between ante-, peri-, and postmortem injuries to the bone.

## 1. Introduction

Bone tissue has an increased capacity for regeneration and adaptation after traumatic injuries. This fundamental property has implications not only for clinical medicine but also for forensic science/medicine. Forensic specialists (especially anthropologists) often have the bone as the only available tissue for a complex forensic analysis, needed to establish the species, causes of death, age, sex, postmortem interval and so on. Bone regeneration is conducted through a complex interplay of cellular, molecular, and mechanical factors, often associated with external elements that can be either temporary (surgical or non-surgical interventions) or permanent (the use of prosthetic materials). The concept of bone plasticity encompasses the dynamic nature of bone tissue, which continuously undergoes remodeling through a coordinated action of bone-forming osteoblasts and bone-resorbing osteoclasts [1,2]. This process is dramatically amplified after traumatic injuries, when the normally homeostatic balance shifts toward repair and regeneration. The molecular determinants governing this transition involve sophisticated signaling cascades, growth factors, and cellular communication systems that have only recently begun to be fully elucidated through advances in molecular biology and tissue engineering research [3,4,5,6].

The cellular component of bone regeneration involves the coordinated activity of multiple cell types, each contributing specific and often unique functions to the healing process. Mesenchymal stem cells serve as the primary progenitor cells for bone regeneration, as they can differentiate into osteoblasts, chondrocytes, and other supporting cell types [7,8]. Immune cells have long been known to have essential functions, both in the early and reparatory phases of healing. For example, the transition from pro-inflammatory M1 macrophages to anti-inflammatory M2 macrophages appears to be crucial for successful bone regeneration, as M2 macrophages promote angiogenesis and tissue repair [9,10]. Angiogenesis represents another critical cellular process in bone regeneration—the coupling of bone formation and neovascularization ensures an adequate nutrient supply and waste removal during the intensive metabolic processes of bone healing. Growth factors such as VEGFs and their interactions with bone-forming cells create a coordinated response, which optimizes the healing environment [10,11].

The temporal progression of bone healing follows a well-characterized sequence of events, which can be monitored through various molecular markers and imaging techniques [12,13]. The initial inflammatory phase, lasting for about one to two weeks, is characterized by hematoma formation, inflammatory cell infiltration, and the release of cytokines and growth factors [14]. The soft callus formation phase usually occurs 2–6 weeks after injury. It involves the differentiation of MSCs into chondrocytes and the formation of cartilaginous tissue, which serves as a template for bone formation. In this phase, there is an overexpression of cartilage-specific markers such as type II collagen or aggrecan, along with the continued expression of growth factors that promote cellular proliferation and differentiation [15,16,17]. The hard callus formation typically occurs during weeks 6–12 and involves the replacement of cartilaginous tissue with mineralized bone. This phase is marked by the expression of bone-specific markers such as osteocalcin, alkaline phosphatase, and type I collagen, along with the activation of mineralization processes [18]. The final remodeling phase, which may continue for months to years, involves the reshaping of the newly formed bone to restore its original structure and mechanical properties [1,18].

The molecular understanding of bone plasticity and regeneration has potentially significant implications in forensic sciences. They were not extensively studied and implemented in practical, forensic environments. However, they have potential uses in areas, such as the interpretation of skeletal trauma, the estimation of the post-traumatic intervals, the postmortem interval, or the differentiation between ante-, peri-, and postmortem injuries to the bone.

## 2. Molecular Mechanisms Involved in Bone Regeneration and Plasticity

Bone regeneration is one of the most remarkable examples of tissue plasticity in the human bone, involving a complex orchestration of cellular interactions, signaling pathways, and molecular mechanisms, activated after trauma but continuing even after the surgical, reconstructive intervention, being highly dependent on the materials used for repair.

### 2.1. Master Transcription Factors Involved in Osteogenic Differentiation

Runt-related transcription factor 2 (Runx2) is a master regulator for bone development and regeneration, being essential in turning stem cells into osteoblasts [19,20], which are needed for the production of bone matrix proteins and activate the bone matrix protein genes [20,21,22], increase the bone mineral density and new bone formation [21,23], and respond to mechanical and biochemical signals by integrating signals from pathways such as Wnt, FGF, Hedgehog [20,22,24]. Runx2 expression follows a distinctive temporal pattern during osteogenesis, being weakly expressed in uncommitted mesenchymal cells, then upregulated in preosteoblasts, reaching maximal levels in immature osteoblasts, and it is downregulated in mature osteoblasts [20,25,26,27]. This dynamic expression reflects its dual-functional nature, namely promoting osteoblast differentiation and inhibiting terminal osteoblast maturation. Its molecular mechanisms involve direct transcriptional regulation of numerous osteogenic genes and coordination with multiple signaling pathways. Runx2 is phosphorylated and activated by the mitogen-activated protein kinase (MAPK) pathway, which can be stimulated by the binding of type I collagen to α2β1 integrins from the surface of the osteoblasts or through treatment with osteogenic growth factors such as FGF2 [28]. It also directly regulates the expression of significant genes, including Col1a1, Spp1 (osteopontin), Ibsp (bone sialoprotein), Bglap2 (Osteocalcin), or Fn1 (fibronectin), during the early stages of osteoblast differentiation [25,29,30,31]. Additionally, it enhances the proliferation of osteoblast progenitors by directly regulating the genes for fibroblast growth factors 2 and 3 (Fgfr2 and Fgfr3), establishing positive feedback loops, which can maintain the osteoprogenitor cell pool [32]. Runx2 activity is enhanced by factors stimulating specific transduction pathways, including BMP2 (which signal through Smad proteins), PTH/PTHrP (signaling through PKA/PKP pathways), and canonical Wnt signaling [28,33,34].

Osterix (Sp7) is another essential transcription factor, necessary for the differentiation of osteoblasts, functioning downstream from Runx2. Runx2 directly regulates Sp7 expression, as suggested by the fact that osteoblasts and bone formation are entirely absent in Sp7-deficient mice [25,32,35,36]. Between Runx2 and Sp7, there is a critical temporal difference—while Runx2 is expressed in Sp7-deficient mice, the converse is false, confirming Runx2′s position as an upstream regulator for osteoblast differentiation [25,35]. Sp7 participates in a positive feedback loop with Runx2, as it activates an osteoblast-specific enhancement of Runx3, contributing to the maintenance of Runx2 expression in differentiating osteoblasts [25,37,38].

### 2.2. Bone Morphogenic Protein Signalling

The BMP signaling pathway is one of the most potent osteoinductive systems for bone regeneration, with an increased capacity to induce ectopic bone and cartilage formation when applied in vivo [39]. It functions through a highly conserved signaling mechanism involving a heterotetrameric serine/threonine kinase receptor with type I and type 2 receptor subunits [40,41,42]. Its signaling cascade starts when BMP ligands bind to the receptor complex, causing the constitutively active type II receptor to phosphorylate and activate the type I receptor, which further phosphorylates receptor-mediated Smad proteins (Smad1, 5, and 8) at discrete C-terminal sites [39,43,44,45]. After phosphorylation, these BMP-specific Smads form complexes with the coSMAD protein Smad4 and then translocate to the nucleus, where they regulate the transcription of genes involved in osteoblast differentiation and bone formation [46,47,48]. The interaction between BMP signaling and Runx2 is a critical convergence point in osteoblast differentiation. BMP signals sent through Smad proteins enhance Runx2 activity, while Runx2 itself can be directly activated through BMP signaling [28]. This synergistic approach explains the potent osteoinductive capacity of BMPs and their critical clinical utility in bone regeneration applications.

Another essential signaling pathway for bone regeneration is canonical Wnt/β-catenin, which enhances the differentiation of mesenchymal stem cell precursors into osteoblasts through β-catenin-dependent mechanisms [49]. The exact pathway suppresses bone resorption by regulating the RANKL (receptor activator of nuclear factor-κB ligand)/osteoprotegerin (OPG) ratio in mature osteoblasts, creating a dual anabolic effect that causes a net increase in bone mass [49]. The molecular mechanism through which Wnt-signaling works involves the stabilization of β-catenin and nuclear translocation, where it acts like a transcriptional co-activator [50]. Without Wnt signals, β-catenin is targeted for degradation by a destruction complex containing glycogen synthase kinase 3β [50]. Studies on mice have shown that the conditional deletion of β-catenin in osteoblast progenitors has led to severely impaired bone formation. At the same time, the constitutive activation of the pathway causes increased bone mass [51,52]. Non-canonical Wnt signaling operates independently of β-catenin and has distinct effects on bone formation and resorption [53]. The non-canonical ligand Wnt5a has been shown to increase RANKL-induced osteoclast formation in mouse macrophage cultures, promoting bone resorption rather than bone formation [54]. It also contributes to osteogenic differentiation through a suppression of the PPARy expression by Wnt5a, inactivating adipogenic differentiation and allowing osteogenic differentiation to proceed [54,55,56,57].

Also involved mainly in osteoclast differentiation and bone resorption is the RANKL/RANK/OPG system. The RANS signaling pathways start with RANKL-induced trimerization of RANK receptors and the recruitment of adaptor proteins (especially TRAF6) to the cytoplasmic tail of RANK [58]. TRAF6, in turn, stimulates multiple downstream signaling pathways, including nuclear factor κB (NF-κB), activator protein-1 (AP-1), and MAPKs [58,59]. These early signaling events lead to the activation of nuclear factor of activated T-cells cytoplasmic 1 (NFATc1), which acts as a master transcription factor for osteoclast differentiation [60,61]. The temporal regulation of RANK signaling involves distinct phases—early RANK-TRAF6 signaling activates transcription factors, intermediate co-stimulatory signals induce calcium oscillations through the Cy2 phospholipase, and late-stage NFARc1 activation drives the expression of osteoclast-specific genes, which are responsible for cellular fusion and bone resorption [58].

Osteoprotegerin (OPG) acts like a soluble decoy receptor for RANKL, being the main endogenous inhibitor of osteoclast formation. The RANKL/OPG ratio is a critical determinant for bone mass, with decreased ratios causing reduced osteoclast activity and increased bone formation [62]. Mechanical stimulation has been shown to decrease RANKL/OPG ratios, providing a mechanism through which mechanical loading promotes bone formation, an aspect that may be further used in clinical applications [63,64].

### 2.3. Growth Factors and Cytokines

Transforming growth factor-β (TGF-β) is an abundant growth factor in bone tissue, with concentrations reaching 200 µg/kg, bone being its largest reservoir in the body [65]. It exists in multiple isoforms, TGF-β1 and TGF-β2 being the predominant forms found in bones. Its effects on bone cells are highly context dependent, with considerable variations depending on the cell type, differentiation stage, and local environmental conditions. TGF-β stimulates the synthesis of major bone matrix proteins, including type I collagen, plasminogen activator inhibitor, and fibronectin, while having differential effects on various cell populations [66]. It acts as a coupling factor, linking bone resorption with bone formation, as it is released from the bone matrix during osteoclastic resorption and subsequently acts on osteoblasts to stimulate bone formation [67,68,69]. Studies on transgenic mice have shown it controls the bone elastic matrix, hardness, and mineral concentration, contributing directly to bone quality and resistance to mechanical loading [69,70,71].

Vascular endothelial growth factor (VEGF) influences skeletal development and postnatal bone repair through multiple mechanisms, including the promotion of blood vessel formation, direct effects on osteoblast function, and the coordination of coupling between angiogenesis and bone formation [72,73]. It is also involved in bone repair through inflammation, endochondral ossification, intramembranous ossification, and bone remodeling phases [72]. It has both paracrine effects (mainly on endothelial cells) and autocrine effects (mainly on osteoblasts), being directly involved in osteoblast differentiation and survival [73]. It has a therapeutic effect on bone regeneration, which has been demonstrated in various animal models. Local VEGF treatment stimulates bone repair by increasing callus vascularity, enhancing bone mineral density and promoting calcified callus formation. However, the dosage should be tightly regulated, and it can stimulate both vascular invasion and osteogenic commitment [74]. Therefore, from a therapeutic perspective, the VEGF dose should be fine-tuned to ensure accelerated angiogenesis and to improve bone formation [74].

### 2.4. Mechanotransduction and Osteocyte Function

Osteocytes act as primary mechanosensory cells in the bone tissue, detecting mechanical stimuli and translating them into biological signals, which further regulate bone formation and resorption [75]. A key mediator in this regard is Sclerostin (SOST); mechanical loading decreases SOST expression, thereby removing Wnt signaling and promoting bone formation [76,77]. Τhe mechanotransduction pathway involving SOST involves multiple mechanisms. Fluid shear stress is known to dose-dependently increase Kindlin-2 expression and decrease SOST expression by downregulating Smad2/3 signaling in osteocytes [75]. The response is abolished if Kindlin-2 is deleted, demonstrating its critical role in mechanotransduction. Slerostin-deficient osteocytes lose the ability to respond to mechanical stimulation [75,78]. This effect has also been shown in clinical environments, as increased SOST serum levels were shown to be associated with bone formation and resorption markers in subjects with immobilization-induced bone loss [79].

Mechanically sensitive ion channels (MSICs) from osteocytes respond to mechanical stimulation by opening as response to membrane tension. The mechanosensing ion channel Piezo1 facilitates the ion exchange between cells and the extracellular environment, leading to the activation of voltage-sensitive calcium channels. Through this, it modulates the intracellular calcium levels and activates downstream signaling pathways, such as Akt-SOST [62]. Loading-induced calcium oscillations release signaling molecules, such as nitric oxide, prostaglandin E2, insulin-like growth factor-1 or β-catenin, which are essential for osteocyte viability and anabolic bone effects [80].

### 2.5. Matrix Metalloproteinases and Extracellular Matrix Remodeling

Matrix metalloproteinases (MMPs) have essential roles in bone regeneration through the regulation of extracellular matrix remodeling, which is needed for both bone formation and resorption [81,82]. MMP-2 influences bone formation by influencing osteoblast and osteoclast proliferation and activity. MMP-2 knockout mice have an altered expression of osteopontin and bone sialoprotein, involved in regulating osteoclast activity and osteoblast development, respectively [83,84]. MMP2 also affects angiogenesis by allowing pericytes to detach, endothelial cells to migrate, and stimulating the secretion of proangiogenic growth factors such as VEGF, FGF-β, and TGF-β [85,86]. It is involved in the degradation of collagen and other matrix proteins during bone remodeling, facilitating bone resorption and the preparation of the matrix for new bone formation [81,82]. MMP-13 is required for the transition from cartilage to bone at growth plates, cleaving type II collagen and aggrecan, which allows for the invasion of blood vessels and the replacement with bone tissue [87]. It also contributes to the proteolytic shedding of lipopolysaccharide receptor CD 14 (together with MMP-12) [88], therefore being involved in the innate host defense, a critical issue in bone repair, as most interventions in this regard are associated with an increased infection risk (mainly due to the trauma, leading to the need for bone reconstruction). In the mature bone, MMP-13 functions in osteoblasts to regulate trabecular bone mass through a continuous remodeling process [89,90].

The interaction between MMPs and extracellular matrix components is known to create feedback loops that regulate stem cell differentiation and bone formation. MMP-13 remodeling of the type I collagen matrix stimulates osteogenic differentiation of human mesenchymal stem cells through an MMP13/integrin α3/Runx2 positive feedback loop [84,91,92,93]. The balance between MMP activity and inhibition determines the rate and extent of matrix remodeling, with disruption of this equilibrium leading to pathological bone loss or inadequate remodeling [81].

### 2.6. Mineralization and Matrix Formation

The mineralization process in the bone mainly involves the formation of hydroxyapatite crystals within an organic matrix, composed primarily of type I collagen [94]. The mineral component has a unique geometry, being represented by crystals not exceeding 30 to 50 nm in length and about 2 nm in thickness [95,96]. Recent studies suggest that the mineralization process proceeds through a non-classical pathway, involving amorphous calcium phosphate (ACP) as a precursor phase, which then transforms into crystalline hydroxyapatite [94,97]. This occurs through a step-growth mechanism where step dimensions correspond to theorized pre-nucleation clusters [94], and is facilitated by collagen I fibrils, which can prevent the self-aggregation of ACP precursor nanoparticles at low supersaturation, thereby promoting a heterogeneous nucleation process [97]. Matrix vesicles, formed and released from osteoblasts, serve as initial sites for mineral nucleation. Current models suggest that hydroxyapatite is initially nucleated within these vesicles and, as crystallites grow larger, they break the membrane of the vesicle and are exposed to the extracellular environment. The initial mineral formation occurs under cellular control, while subsequent mineral propagation is controlled by collagen and other matrix proteins [98,99,100].

Non-collagenous proteins, such as osteocalcin, osteopontin, or bone sialoprotein, also play critical roles in bone mineralization by controlling crystal nucleation, growth, and organization. Osteocalcin, which contains three γ-carboxy-glutamic acid residues, binds calcium and modulates its metabolism by mediating the association with hydroxyapatite [101,102,103]. The protein also functions as an inhibitor for bone mineralization, with osteocalcin-null mice showing larger hydroxyapatite crystal sizes [104,105]. Phosphorylated proteins are also critically involved in mineralization control, their importance being demonstrated by the severe mineralization that occurs in instances in which their function is disrupted [106,107,108].

## 3. Practical Forensic Applications of the Molecular Mechanisms of Bone Regeneration

### 3.1. Establishing Forensically Relevant Time Intervals

The molecular mechanisms of bone regeneration provide forensic practitioners with highly specialized tools that may be able to distinguish between antemortem, perimortem, and postmortem injuries. This topic was initially researched using histological methods—see de Boer et al., who assessed the posttraumatic time interval in human dry bone [109,110]. The expression patterns for key regulatory molecules, such as Runx2, osteocalcin, or BMP, are known to follow predictable temporal sequences during bone healing. The temporal dynamics for Runx2 expression detected using immunohistochemistry were shown to increase in healing bone tissue (calvaria and tibial) up to three weeks post-fracture, with a large number of Runx2-positive cells scattered through the trabecular bone [111]. The number was significantly decreased after five weeks [111], and four months after injury, the Runx2 expression was close to 0 [112]. These results suggest that an immunohistochemical analysis of Runx2 may be used to date relatively recent injuries. These results were confirmed by studies on human osteoblast cultures, which showed that in the first seven days, the Runx2 expression is weakly expressed in uncommitted cells; the peak expression is obtained after 7–14 days, in immature osteoblasts, while between 14 and 28 days, the Runx2 expression decreases as osteoblasts mature [113].

**Figure 1:**
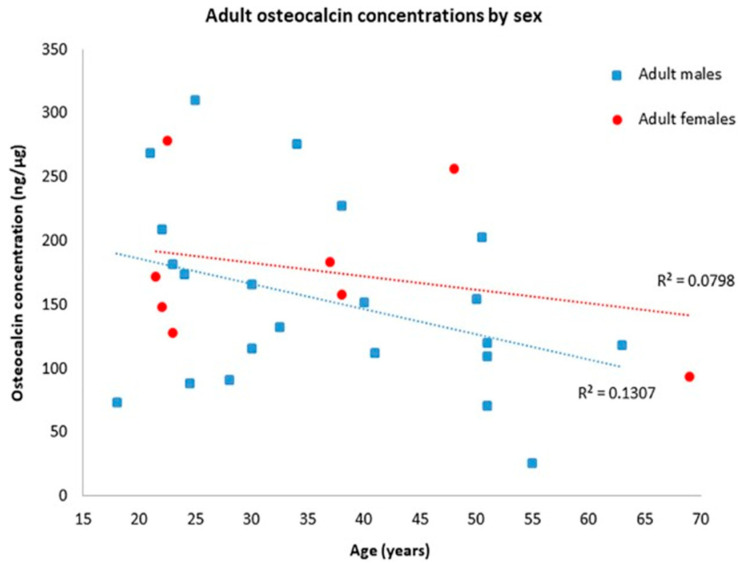
Distribution of adult osteocalcin concentrations divided by sex. From Scott et al. Comparing biological and pathological factors affecting osteocalcin concentrations in archaeological skeletal [114].

Osteocalcin also demonstrates a distinct temporal expression but significantly different from Runx2. In the first 14 days, during the initial inflammatory and proliferative phases, the osteocalcin expression is minimal. The peak expression is obtained between 14 and 28 days, which coincides with the mineralization phase. The expression remains raised after 28 days (up to at least four months), through the mineralization and remodeling phases [113]. Therefore, osteocalcin is particularly useful for identifying more advanced healing stages, even after the Runx2 expression declines. Osteocalcin is very stable over long periods. For example, Scott et al. studied 46 skeletons from the Danish Sct. Albert’s cemetery (around 1250–1536), which exhibits a notable difference in the distribution of concentrations between males and females, with increased variability attributed to pathological conditions characterized by bone remodeling [114] (see Figure 1). Ritz et al. found the S-aspartic acid content of purified bone osteocalcin to have a close relationship with the age at death, suggesting it may be used as an aging peptide of the organic bone matrix [115].

BMPs (especially BMP-2) have also been studied as a potential marker for bone dating in its association with bone remodeling. BMP-2 increases rapidly after trauma (in the first three days), as part of the initial healing cascade; the peak activity is reached between 3 and 14 days, coinciding with osteoclast recruitment and differentiation. Between 14 and 28 days, the signaling continues to be sustained, to support ongoing bone formation and mineralization. Still, the expression starts to decline, while after the eighth week, the values return to the baseline [116,117,118]. The BMP-2 expression is spatially localized in areas of active bone formation, such as periosteum and callus, and delayed or reduced expression is associated with impaired or delayed bone healing [119,120]. Using murine models, Cho et al. found that BMP-2 has maximal expression on day 1 after fracture; BMP-3, -4, -7, and -8 have a restricted period of expression from day 14 to day 21 after fracture, during cartilage formation. At the same time, BMP-5 and -6 were expressed from day 3 to 21 [118]. These animal models must, before entering forensic practice, be validated on human/autopsy studies.

VEGF expression peaks at one to three weeks after fracture and remains elevated throughout the whole healing process, emphasizing its critical role in angiogenesis during the proliferative phase of bone healing [72,121]. Micro-CT angiographic studies reveal that mRNA VEGF expression follows a consistent pattern during fracture healing, with an exponential rise in week 2, followed by a decline in week 3. The number of branches, total volume, and diameter of blood vessels that are identifiable in micro-CT angiography show parallel patterns to VEGF expression [121]. Quantitative VEGF analysis provides established forensic applications for estimating the age of a wound, especially in the 7-day threshold determination, with VEGF-positive ratios exceeding 50% suggesting a wound older than 1 week [122]. VEGF expression during bone healing involves multiple cellular sources and signaling pathways, which can be analyzed forensically. Hypertrophic chondrocytes secrete high levels of VEGF during enchondral ossification, while osteoblasts and inflammatory cells increase it during intramembranous ossification [72]. The molecular mechanisms include hypoxia-induced VEGF expression in the surrounding bone and inflammatory cells [123,124,125], VEGF sequestration in the fibrin matrix/surrounding hematoma following injury, creating a reservoir of VEGF that can be detected using histological and molecular methods [72,126,127]. VEGF is one of the most studied temporal markers on bone tissue, being validated in larger samples. For example, Hayashi et al. found that >50% immunopositive VEGF cells are highly indicative for a survival of at least one week [122].

TGF-β1 expression is moderately increased (around 1.7-times compared to baseline) at seven days post-fracture and returns to near baseline levels thereafter [128]. Similarly, in murine models, TGF-β2 and TGF-β3 exhibit maximal expression at day seven, coinciding with the expression of type II collagen during cartilage formation [118]. Protein localization studies show that TGF-β1 is mainly localized within inflammatory cells and the extracellular matrix within the fracture gap during the early healing period (day three), mostly to the osteotomized bone edges and periosteum (by day seven) and within the newly formed bone matrix during the remodeling phase [128]. As TGF-β1 is expressed mainly in the inflammatory cells in the first days, it may be used to distinguish between antemortem and postmortem injuries. Similarly, TGF-β receptors have predictable temporal patterns. TGF-β receptor II showed an initial downregulation, followed by upregulation, reaching peak levels at day nine after injury (1.8-times higher compared to the baseline) [128]. Its localization may provide additional forensically relevant data: on day three, TGF-β receptor II is mainly expressed in inflammatory and periosteal cells; by day seven, in osteoblast and mesenchymal cells at the bone edges; and by day 37, mainly in osteocytes and osteoblasts in the remodeling bone [128].

MMPs also have distinct temporal expression patterns. In the first few days, lower serum MMP-2 levels are seen in patients with successful bone healing after the surgical intervention, higher levels being shown as predictors for poor healing [129]. Two days after the intervention, the MMP-9/MMP-2 ratio is significantly higher in successful healers, emphasizing a distinct early MMP-2 profile associated with better regeneration. The MMP-9 to MMP-2 ratio remains a valuable indication for the healing process up to four weeks after intervention [129]. The results’ usefulness for forensic practice is limited by the low number of subjects on which it was studied. Persistently high or overexpressed MMP-2 is linked with poor or delayed bone healing, making it a potential negative prognostic biomarker [129,130]. For the estimation of postmortem interval, however, MMP-2 and MMP-9 have limited usefulness, due to the variability in MMP expression across evaluated samples and the high variability in individual limits [131].

In addition to bone, cartilage postmortem time identification has been evaluated by some researchers. For example, Alibegovic et al. processed forty-five articular cartilage sections with five routine stains and then extracted mean gray values after color deconvolution in ImageJ; linear regression between these objective intensities and the modified Bern visual scores yielded high correlation coefficients for the evaluated stains, with the highest—an r of 0.93—for Massson trichrome, suggesting its potential usefulness for PMI on cartilage tissue [132]. A controlled porcine burial experiment has shown a slow decline in chondrocyte viability during the first three days, followed by an exponential decline to 16% on buried models and 5% on surface models by day ten, with accelerated decay at higher ambient temperatures and in shallower burial sites [133]. Another pilot study on human samples showed that knee cartilage stored at 22 °C reduced its viable fraction by relatively 50% by day 8 and continued to fall significantly after day 9, across 4–35 °C storage curves, positioning mid-single-digit days as a practical upper limit for viability-based PMI [134].

### 3.2. Species and Individual Identification

Sometimes, in forensic anthropology, a highly relevant question that must be asked, before going further with the scientific analysis, is whether a particular bone or part of the bone is human or not. Even if, in most cases, the question has an easy answer for most anthropologists, sometimes the species identification is hardened by the quantity of obtained bone or its degradation [135,136]. Molecular bone markers have a remarkable utility in species identification, especially in these circumstances. The COL1A2 protein barcode methods have been shown to have 100% accuracy in species identification across 30 vertebrate species, by analyzing the collagen type 1 alpha two sequences [137].

Molecular markers can reveal pathological conditions that may have contributed to fracture susceptibility or healing complications. Bone turnover markers provide insights into metabolic bone disease such as osteoporosis, characterized by increased β-CTX and osteocalcin levels, suggestive of increased bone resorption and turnover [138]. The results have been confirmed on a relatively large cohort, but their forensic usefulness must be further assessed [138]. Metabolic profiling, through the ForensicOMICS approach, which combines metabolomics, lipidomics, and proteomics, can provide a comprehensive assessment of bone health status and, therefore, identify metabolic dysregulation that may influence bone strength and healing capacity [139].

### 3.3. Trauma Mechanism Reconstruction

Molecular markers can aid in the reconstruction of the mechanism of trauma by providing information about the distribution of forces and conditions involved in the appearance of the fracture. The analysis of inflammatory markers and their spatial distribution within the bone tissue may suggest the direction and magnitude of applied forces. Dynamic loading may cause a weakening of the bone materials by structural microdamage [140]. Various studies have shown that bone microcracking can be repaired through bone remodeling, a cellular process in which the damaged bone is resorbed through osteoclastic activity, which is followed by new tissue generation by osteoblasts [141,142]. The process of remodeling is conducted by mechanosensitive osteocytes, through a system of intercommunicating canaliculi, which acts as transducers [143,144]. Mechanical stimulation of mesenchymal stem cells induces CXCL1 and CCL2, leading to the recruitment of classical monocytes, which can further be differentiated into bone-resorbing osteoclasts, therefore controlling site-specific localization of bone inflammation and damage [145]. The initial results, obtained from animal models, seem promising, and a further translation into forensic pathology is relatively easy to perform. MMP-3 was shown to be significantly correlated with transformed fracture energy and IL-1β, with the overall Injury Severity Score [146] further emphasizing the potential usefulness of molecular biomarkers to trauma mechanism reconstruction.

### 3.4. Taphonomic Assessment

The analysis of protein preservation patterns may bring useful information about burial conditions, environmental exposure, and diagenetic changes. The differential preservation in bone tissue is fundamentally linked to their mineral binding properties, which create a protective microenvironment that restricts microbial decomposition and enzymatic degradation, allowing for the reconstruction of the molecular components, even hundreds of millions of years later [147,148]. Studies on fossil specimens have shown that proteins within bones may survive through diagenesis, despite complex histories of chemical alteration, attesting to their remarkable resilience [147,148]. Recent studies have shown that oxidizing conditions in the first 48 h after death most likely promote molecular stabilization reactions, which are critical for long-term protein preservation by creating cross-linking and binding patterns that enhance molecular stability [148]. The role of microorganisms in bone diagenesis has been extensively studied, revealing complex interactions between bacterial activity and protein preservation. Both gut and soil bacteria play key roles in bone diagenesis and protein decay on relatively short timescales [149]. The relevant biomolecules have also been extensively studied in the last few years through large-scale analyses, using both human bones from casework and cemeteries, as well as animal proxies [149,150,151,152]. The mineral matrix acts as a physical barrier that limits microbial access to organic components. In environmental conditions that cause an accelerated loss of the mineral matrix, such as acid environments, the microbial access is eased, and the degradation rate is increased [153,154,155].

#### Limitations of This Study

As this is a narrative review, the main limitation lies in the selection of articles used as the backbone. The PMI should always be evaluated in practical contexts with age-related reduced osteogenic markers, as well as associated diseases (especially osteoporosis and diabetes, both of which are known to delay callus maturation). Additionally, postmortem changes (such as autolysis, putrefaction, embalming, and temperature-related) can affect antigenicity, potentially decreasing the accuracy of the results in practical environments. Contamination is another potential confounding factor—cross-contamination between samples, environmental contamination, human contamination from laboratory personnel, and instrumental contamination from inadequately sterilized equipment. Most molecular marker studies lack a sufficient sample size and a broad-enough age range to develop robust predictive models. Therefore, similar studies should be evaluated, using complex analytical/statistical models, to increase their practical usefulness. Even in the more precise/accurate studies, postmortem interval estimation shows substantial imprecision.

## 4. Conclusions

Bone regeneration involves a sophisticated orchestration of cellular, molecular, mechanical and environmental factors, with master transcription factors such as Runx2 and Osterix serving as key regulators. Their potential forensic usefulness has been less studied. However, there are numerous potential applications, many only hypothesized, which include time interval estimation, species identification, trauma reconstruction and taphonomic/archeological uses.

## Data Availability

No new data were created.

## Abbreviations

ACP	Amorphous Calcium Phosphate
AP-1	Activator Protein-1
BMP	Bone Morphogenetic Protein
BMP-2	Bone Morphogenetic Protein-2
COL1A2	Collagen Type 1 Alpha 2
Col1a1	Collagen Type 1 Alpha 1 gene
CXCL1	C-X-C Motif Chemokine Ligand 1
CCL2	C-C Motif Chemokine Ligand 2
FGF	Fibroblast Growth Factor
FGF2	Fibroblast Growth Factor 2
Fgfr2	Fibroblast Growth Factor Receptor 2 gene
Fgfr3	Fibroblast Growth Factor Receptor 3 gene
Fn1	Fibronectin 1 gene
Ibsp	Integrin Binding Sialoprotein gene (bone sialoprotein)
IL-1β	Interleukin-1 Beta
MAPK	Mitogen-Activated Protein Kinase
MMP	Matrix Metalloproteinase
MMP-2	Matrix Metalloproteinase-2
MMP-3	Matrix Metalloproteinase-3
MMP-9	Matrix Metalloproteinase-9
MMP-12	Matrix Metalloproteinase-12
MMP-13	Matrix Metalloproteinase-13
MSCs	Mesenchymal Stem Cells
MSICs	Mechanically Sensitive Ion Channels
NFATc1	Nuclear Factor of Activated T-cells Cytoplasmic 1
NF-κB	Nuclear Factor κB
OPG	Osteoprotegerin
PKA	Protein Kinase A
PKP	Protein Kinase P
PPARγ	Peroxisome Proliferator-Activated Receptor Gamma
PTH	Parathyroid Hormone
PTHrP	Parathyroid Hormone-related Protein
RANK	Receptor Activator of Nuclear Factor κB
RANKL	Receptor Activator of Nuclear Factor κB Ligand
Runx2	Runt-related Transcription Factor 2
Runx3	Runt-related Transcription Factor 3
SMAD	Mothers Against Decapentaplegic homolog proteins
SOST	Sclerostin gene
Sp7	Specificity Protein 7 (Osterix)
Spp1	Secreted Phosphoprotein 1 gene (osteopontin)
TGF-β	Transforming Growth Factor-β
TGF-β1	Transforming Growth Factor-β1
TGF-β2	Transforming Growth Factor-β2
TGF-β3	Transforming Growth Factor-β3
TRAF6	TNF Receptor Associated Factor 6
VEGF	Vascular Endothelial Growth Factor
β-CTX	β-CrossLaps (C-terminal telopeptide of type I collagen)
Wnt	Wingless-related integration site signaling pathway
Wnt5a	Wingless-related integration site 5a
